# Supplementary material for: Identifying prostate cancer and its clinical risk in asymptomatic men using machine learning of high dimensional peripheral blood flow cytometric natural killer cell subset phenotyping data
Source: eLife. 2020 Jul 28;9:e50936. doi: 10.7554/eLife.50936 (PMC7386909; doi:10.7554/eLife.50936)
Supplement: Transparent reporting form [file elife-50936-transrepform.docx]

***eLife’s* transparent reporting form**

We encourage authors to provide detailed information *within their submission* to facilitate the interpretation and replication of experiments. Authors can upload supporting documentation to indicate the use of appropriate reporting guidelines for health-related research (see [EQUATOR Network](http://www.equator-network.org/%20)), life science research (see the [BioSharing Information Resource](https://biosharing.org/)), or the [ARRIVE guidelines](http://www.plosbiology.org/article/info:doi/10.1371/journal.pbio.1000412) for reporting work involving animal research. Where applicable, authors should refer to any relevant reporting standards documents in this form.

If you have any questions, please consult our Journal Policies and/or contact us: [editorial@elifesciences.org](mailto:editorial@elifesciences.org).

**Sample-size estimation**

- You should state whether an appropriate sample size was computed when the study was being designed
- You should state the statistical method of sample size computation and any required assumptions
- If no explicit power analysis was used, you should describe how you decided what sample (replicate) size (number) to use

Please outline where this information can be found within the submission (e.g., sections or figure legends), or explain why this information doesn’t apply to your submission:

Information about data collection can be found under subsection Data Collection under the section Materials. We profiled natural killer (NK) cell subsets in the blood of 72 asymptomatic men with Prostate Specific Antigen (PSA) levels <20 ng/ml, of whom 31 had benign disease (no cancer) and 41 had prostate cancer. Hence we have: Group 1: 31 patients with benign disease (no cancer) and Group 2: 41 patients with prostate cancer.

Statistical power is the probability that the test rejects the null hypothesis when a specific alternative hypothesis is true. We used the Power of T-test to determine whether we had sufficient samples for testing our hypotheses. Given a significance of 0.05 we explore the change in sample size between 2 and 100 with low (es=0.2), medium (es=0.5), and high (es=0.8) effect sizes, and with a ratio of 1 and 2, in Figures 1 and 2 respectively. Ratio is the number of observations in sample 2 relative to sample 1. Within the Figures, multiple curves are plotted to show the impact on statistical power.

Figure 1 shows the “Power of T-Test curve a=0.05, ratio=1.” The graph shows the relationship between sample size and power. The sample size (n=26) that would be necessary to achieve 80% power with a significance of 0.05 and 0.01 with a high effect size (es=0.80) are shown below.

a=0.05, ratio 1

- Required sample size for group 1: 26

- Required sample size for group 2: 26

Total sample size: 52. Therefore our sample size of Group 1: 31 and Group 2: 41 is sufficient. We have more samples in each group than those required to reliably test our hypothesis.

Figure 1: a=0.05, ratio=1

Our sample is also sufficient for a ratio of 2. So,

a=0.05, ratio 2

- Required sample size for group 1: 19

- Required sample size for group 2: 38

Total sample size: 57. Therefore our sample size is sufficient. We have more samples in each group than those required to reliably test our hypothesis.

Figure 2: a=0.05, ratio=2

In the paper, we report results of statistical tests, and we can assume that the hypotheses can be correctly accepted or rejected since the sample sizes are appropriate.

**Replicates**

- You should report how often each experiment was performed
- You should include a definition of biological versus technical replication
- The data obtained should be provided and sufficient information should be provided to indicate the number of independent biological and/or technical replicates
- If you encountered any outliers, you should describe how these were handled
- Criteria for exclusion/inclusion of data should be clearly stated
- High-throughput sequence data should be uploaded before submission, with a private link for reviewers provided (these are available from both GEO and ArrayExpress)

Please outline where this information can be found within the submission (e.g., sections or figure legends), or explain why this information doesn’t apply to your submission:

- As per standard practice, flow cytometric analysis was performed once on each clinical sample, with appropriate controls included in all experimental runs (Page 3, line 119; Figure 1). The flow cytometry experiments have been reported according to the ‘*Minimum Information about a Flow Cytometry Experiment (MIFlowCyt)*’ guidelines, as described by [Lee JA et al., MIFlowCyt: The minimum information about a flow cytometry experiment. Cytometry Part A 2008, 73A: 926-30](https://onlinelibrary.wiley.com/doi/epdf/10.1002/cyto.a.20623);
- Only biological replicates were included (i.e. samples from different individuals attending the Urology Clinic at University Hospitals of Leicester (see Page 2, lines 72-84; page 3, line 85; page 3, Table 1);
- No outliers were encountered;
- No criteria for excluding / including data were applied.

**Statistical reporting**

- Statistical analysis methods should be described and justified
- Raw data should be presented in figures whenever informative to do so (typically when N per group is less than 10)
- For each experiment, you should identify the statistical tests used, exact values of N, definitions of center, methods of multiple test correction, and dispersion and precision measures (e.g., mean, median, SD, SEM, confidence intervals; and, for the major substantive results, a measure of effect size (e.g., Pearson's r, Cohen's d)
- Report exact p-values wherever possible alongside the summary statistics and 95% confidence intervals. These should be reported for all key questions and not only when the p-value is less than 0.05.

Please outline where this information can be found within the submission (e.g., sections or figure legends), or explain why this information doesn’t apply to your submission:

**Statistical analysis methods should be described and justified:** Relevant information has been provided throughout the paper. Application of the various statistical methods used was based on established criteria and practice, and these were justified. Statistical analysis methods have been described and justified throughout the paper:

- ***Materials section:*** Statistical analysis subsection provides descriptive statistics of the dataset and analysis;
- ***Materials section:*** ‘*Identifying Predictors from Immunophenotyping Data using a Genetic Algorithm’* subsection describes how the Genetic Algorithm was applied to identify the best fingerprint for detecting prostate cancer in men with PSA levels<20 ng.ml^-1^. Reports results of statistical analysis;
- ***Results section*** provides detailed analysis of the results including statistical analysis of the findings;
- ***Results section:*** ‘S*tatistically Significant Differences in Predictive Performance when using various Feature Subsets’* subsection discusses the results (a) average AUC values (b), Average Optimal ROC points (TPRs), (c) Average Optimal ROC points (FPRs), (d) Average Accuracy values. A boxplot contains 30 points, where each point is the average performance evaluation value (i.e. AUC, ORP TPR, ORP FPR, Accuracy) from one 10-fold run using the various feature sets of the Friedman's two-way Analysis of Variance (ANOVA) test was used to determine whether statistically significant differences between the mean AUC values across the results obtained by the classifier when using the various subset of features exist. We report the results of *ad hoc* tests, with p values and conference intervals;
- *‘Predicting Low/Intermediate Risk Cancer vs. High Risk Cancer’* section also contains a statistical analysis of the dataset in the “*The cancer patients dataset*” section and the Results section.

**Raw data should be presented in figures whenever informative to do so (typically when N per group is less than 10):** This was not possible, however, we descriptive statistics of the dataset are provided in Table 3.

- **For each experiment, you should identify the statistical tests used, exact values of N, definitions of center, methods of multiple test correction, and dispersion and precision measures (e.g., mean, median, SD, SEM, confidence intervals; and, for the major substantive results, a measure of effect size (e.g., Pearson's r, Cohen's d):** This information and justification for the tests used have been provided throughout.
- **Report exact p-values wherever possible alongside the summary statistics and 95% confidence intervals.** **These should be reported for all key questions and not only when the p-value is less than 0.05:** This information has been provided throughout.

(For large datasets, or papers with a very large number of statistical tests, you may upload a single table file with tests, Ns, etc., with reference to sections in the manuscript.)

**Group allocation**

- Indicate how samples were allocated into experimental groups (in the case of clinical studies, please specify allocation to treatment method); if randomization was used, please also state if restricted randomization was applied
- Indicate if masking was used during group allocation, data collection and/or data analysis

Please outline where this information can be found within the submission (e.g., sections or figure legends), or explain why this information doesn’t apply to your submission:

- Details on the individuals from whom samples were collected are provided in the Materials section (‘*Data Collection*’);
- No allocation to treatment method and/or randomisation was undertaken;
- No masking was used;
- An Ethics Statement has been provided;
- Experimental details on the flow cytometric staining of peripheral blood mononuclear cells (PBMCs) and sample analysis is provided in the ‘*Flow Cytometric Analysis*’ section.

**Additional data files (“source data”)**

- We encourage you to upload relevant additional data files, such as numerical data that are represented as a graph in a figure, or as a summary table
- Where provided, these should be in the most useful format, and they can be uploaded as “Source data” files linked to a main figure or table
- Include model definition files including the full list of parameters used
- Include code used for data analysis (e.g., R, MatLab)
- Avoid stating that data files are “available upon request”

Please indicate the figures or tables for which source data files have been provided:
